# Zolpidem-induced suicide attempt: a case report

**DOI:** 10.1186/2008-2231-21-77

**Published:** 2013-12-20

**Authors:** Sameh Mortaz Hejri, Mehrdad Faizi, Mohammad Babaeian

**Affiliations:** 1 Tehran University of Medical Sciences, Tehran, Iran; 2 Department of Pharmacology & Toxicology, Shahid Beheshti University of Medical Sciences, Tehran, Iran; 3 National Drugs and Poisons Information Center, Deputy of Food and Drug, Shahid Beheshti University of Medical Sciences, Tehran, Iran; 4 Department of Pharmacognosy, School of Pharmacy, Shahid Beheshti University of Medical Sciences, Vali-e-Asr Avenue, Niayesh Junction, P.O. Box: 14155–6153, Tehran, Iran

**Keywords:** Zolpidem, Suicide attempt, Insomnia

## Abstract

Zolpidem is a popular drug indicated for the short-term treatment of insomnia. Side effects are not uncommon with zolpidem. Herein we describe an Iranian 27-year-old man with no known mood disorder or neuropsychological disease who attempted suicide upon taking zolpidem. There are two interesting facts about this case: Firstly, the patient had not history of suicide attempt or thinking. Secondly, this case had experienced suicide ideation after taking 20 mg of zolpidem, suggesting a possible correlation between zolpidem psychological effects and dangerous psychological behaviors.

## Background

Zolpidem is an Imidazopyridine agent indicated for the short-term treatment of insomnia [1,2]. It has gained popularity as an alternative to benzodiazepine for the treatment of insomnia because of its milder and less problematic side effects [3]. Nevertheless, side effects are not uncommon with zolpidem. Hajak and Babdelow in 1998 studied 16,944 cases of zolpidem use and reported adverse events in 1.1% of the cases. The most common reported side effects were nausea, dizziness, malaise, hallucination, nightmares, agitation, and headache [4]. Although zolpidem appears to be well-tolerated in adults and in the elderly, even when administered in accordance with prescribing instructions [5], there are case reports of abuse, dependence, withdrawal syndrome and severe and various adverse effects [6-9]. However, in the majority of these cases, the recommended dose of zolpidem was exceeded and most patients had a history of substance abuse and/or a psychiatric disorders [10]. In 1992, Ansseau et al. [11] reported the cases of 2 patients who developed visual hallucinations and amnesia shortly after the intake of zolpidem. Iruela et al. [12] reported a case of a patient presenting with macropsia without amnesia. therefore, the number of reports of neuropsychiatric reactions associated with zolpidem use has increased, indicating that zolpidem should not be considered risk free, even at therapeutic doses, and might produce transient cognitive and behavioral impairments similar to those caused by benzodiazepines [8,13-15].

Some authors [6,13,16-19] identify 4 factors associated with increased risk of zolpidem-associated psychotic or delirious reactions. One of them is using zolpidem doses of 10 mg or higher.

We present a case of individual in whom using 20 mg of zolpidem was associated with suicide ideation.

## Case presentation

A 27-year-old male, university student, unmarried, dormitory resident with no known mood disorder or neuropsychological disease and abnormal sleep behaviors, had self-referred to a medical toxicologist because of a new experience with zolpidem abuse. He had no history of health compromising or suicidal behaviors and was free of neuropsychological drugs, except for PRN use of zolpidem 5 to maximum of 15 mg during the last year.

In May 2013 he declared that he had no dependence to zolpidem. Despite his continuant use for weeks, he strongly believed that he was not and would not be addicted to zolpidem. He applied it as a sleeping drug at first but after a while, he became interested in the pleasant mental experiences of taking zolpidem even more than its sleeping effect alone. The most important trigger for his continuant abuse of zolpidem, as he said, was his bad emotional status and pressure of thoughts which got severe dramatically at nights. He described his condition by this sentence: “I wanted to forget everything”. Gradually zolpidem could not make him sleep anymore but instead resulted in spending his time doing his favorite things such as listening to music, watching a movie, web surfing or chatting up with friends.

The side effects of taking zolpidem, as reported by him were nausea, vomiting, insomnia, nightmares, dizziness and hangover the next day, headache (very common), right side abdominal pain the next day, visual hallucination, increased body temperature, talkativeness, greasy hair, lack of control on his speech as telling personal secrets to others, feeling a bitter taste in saliva, change of skin color and the smell of body sweat.

One night, after a rush of annoying thoughts, with a great desire for zolpidem, he took two pills of zolpidem. The following day he woke up with in sentiency at 8’o clock in the morning, thought a little to remember the last night’s events, and memorized taking two pills of zolpidem. After a few minutes he noticed the blister of zolpidem by his bed side and saw that 6 of pills are taken, while he was sure that it was a full blister and he had taken just two of them last night. He tried his best to remember exactly that what was happened last night and the only result was suicidal thoughts by zolpidem; After taking 20 mg of Zolpidem at first and beginning the effect of the used zolpidem in a few minutes, he considered taking 4 other pills in order to suicide and did not remember the afterwards.

## Conclusion

Zolpidem has a potential for either medical misuse when the drug is continued long term without or against medical advice, or recreational use when the drug is taken to achieve a “high” [20]. Data from the “Centre for evaluation and information on pharmacodependence” and the 53 literature case reports highlights significant dependence and abuse potential of zolpidem [21].

There are some reports of suicidal attempts by zolpidem, but to the best of our knowledge, this is the first reported case of zolpidem-induced suicide attempt by zolpidem.

In 1996 a 68-year-old female ingested at least 30 tablets of 10 mg Ambien® was found dead at home. Toxicological analyses revealed blood concentration of 4.1, 19.3 and 2.3 μg/ml of zolpidem, meprobamate and carisoprodol, respectively [22]. A study in 1999 on 8 postmortem cases, reported 5 cases of deaths following zolpidem intoxication [23]. In 1999 Gock SB et al. report, the cause of death for two cases was determined to be acute zolpidem overdose, and manner of death was suicide [24].

It seems the patient attempted suicide when he was under influence of the CNS effects of zolpidem. Therefore it might be a high risk of attempting suicide when people with no previous decision for suicide, take zolpidem.

Because this case is based on one personal statement, additional studies and investigations are needed to refute or confirm this side effect, because zolpidem is a widely used drug in the world and Iran.

### Consent

Written informed consent was obtained from the patient for the publication of this report.

## Competing interests

The authors declare that they have no competing interests.

## Authors’ contributions

SMH: writing the primary manuscript and describing the case. MF: language and scientific editing of the manuscript. MB: primary and psychiatric evaluation of the case. All authors read and approved the final manuscript.

## Acknowledgements

We would like to Acknowledge Dr. Ali Tabaeian for his tremendous helps and valuable suggestions.
